# Assessing neurobiology of lifelong premature ejaculation through brain MRI structural similarity gradient

**DOI:** 10.1186/s41747-025-00661-3

**Published:** 2025-12-18

**Authors:** Qiming Deng, Qing Hu, Yan Lei, Xin Zhang, Qingqiang Gao, Xiaozhi Zhao, Yutian Dai, Cong Wang, Jiaming Lu, Bing Zhang

**Affiliations:** 1 Department of Radiology, Nanjing Drum Tower Hospital, Affiliated Hospital of Medical School, Nanjing University, Nanjing, China; 2 Institute of Medical Imaging and Artificial Intelligence, Nanjing University, Nanjing, China; 3 Medical Imaging Center, Department of Radiology, Nanjing Drum Tower Hospital, Affiliated Hospital of Medical School, Nanjing University, Nanjing, China; 4 Department of Andrology, Nanjing Drum Tower Hospital, Affiliated Hospital of Medical School, Nanjing University, Nanjing, China

**Keywords:** Magnetic resonance imaging, Microscale transcriptome profile, Neurotransmitter, Premature ejaculation, Structural similarity gradient

## Abstract

**Objective:**

This study explored the gradient changes in structural similarity based on the cortical structure of patients with lifelong premature ejaculation (LPE) and further analyzed the characteristics of the associations between these changes and clinical phenotypes, gene expression profiles, and neurotransmitter distributions.

**Materials and methods:**

This study employed a novel method, morphological inverse divergence (MIND), to construct structural similarity gradients for 62 LPE patients and 53 healthy controls. Between-group comparisons were performed to examine the abnormalities in gradients among LPE patients. Partial least squares regression analysis explored the relationships between gene expression profiles and gradient changes, as well as neurotransmitter expression associations with these alterations.

**Results:**

We found that both groups showed a classic unimodal-to-cross-modal transition along the principal gradient. In LPE patients, the principal gradient increased in the left visual cortex and right prefrontal regions but decreased in the right cingulate gyrus. The secondary gradient also decreased in the right somatosensory cortex and bilateral visual cortices. Notably, changes in these gradients in the right somatosensory and visual cortices were significantly negatively correlated with clinical phenotypes. Connectome-transcriptome analysis revealed that abnormal gradient patterns were linked to whole-brain gene expression profiles, with enriched genes in pathways related to hormone activity and other functions. Additionally, there was a spatial correlation between the gradients and neurotransmitter densities.

**Conclusion:**

We identified the biological pathways enriched in genes associated with the pathological process of LPE and characterized the distribution patterns of neurotransmitter receptors and transporters, thereby providing critical insights into the neuroimaging and neurobiological underpinnings of LPE.

**Relevance statement:**

The MIND-based brain structural similarity gradient exhibits a pattern of segregation and integration. We analyzed the association between this structural gradient and the clinical phenotype of primary premature ejaculation, offering novel insights into the neuroimaging and neurobiological mechanisms underlying the disorder.

**Key Points:**

- We employed a novel approach, morphometric inverse divergence, to construct the brain structural similarity gradient.
- We provide evidence for abnormal structural similarity gradients in patients with LPE.
- We found an association between abnormal changes in gradients and clinical phenotypes, gene enrichment pathways, as well as neurotransmitter density.

**Graphical Abstract:**

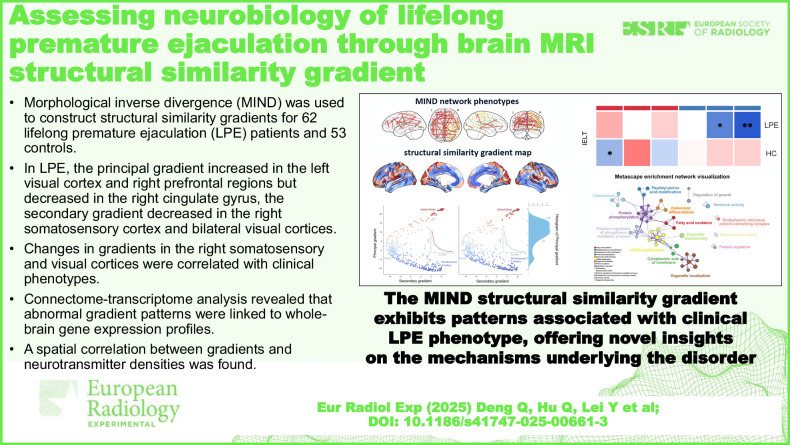

## Background

Ejaculatory dysfunction, especially premature ejaculation, is considered the most common sexual dysfunction in men, affecting approximately 20–30% of men of all ages worldwide [1, 2]. According to the guidelines established by the International Society of Sexual Medicine, lifelong premature ejaculation (LPE) is defined as ejaculation that occurs before or within 1 min after vaginal penetration in every or almost every sexual encounter since the first sexual experience, with a complete or almost complete lack of control over ejaculation, and causing distress and interpersonal difficulties for the patient and/or their sexual partner [3].

However, the pathophysiological mechanism of this disease remains unclear. Current research indicates LPE may involve genetics [4], psychology [5], and neurobiology [6]. Recent studies have focused on neurological mechanisms: Zhang et al identified impaired brain responses and functional integration in LPE patients using functional magnetic resonance imaging (MRI) [7]; Lu et al observed enhanced intra- and inter-network connections in LPE patients, along with dopamine pathway synergism disorders [8, 9]; Zhou et al reported abnormalities in key brain network nodes and connections in LPE patients [10]; Chen et al found left amygdala segregation abnormalities via brain network analysis [11]. Unfortunately, these conclusions from these studies are inconsistent and unstable, as they primarily focus on specific brain regions or circuits while neglecting deeper network structures. Brain gradients can well make up for this deficiency because they provide a characterization of the topographical organization of cortex [12–15].

Being current gradient research predominantly based on resting-state data, here we propose a novel approach: constructing brain structure similarity gradients using a structure similarity network, which was constructed by *morphometric inverse divergence* (MIND) [16]. The MIND network demonstrates a stronger coupling with the transcriptional characteristics of inter-regional similarity, thereby more accurately capturing the relationship between structural MRI similarity and gene transcriptional similarity across cerebral cortical regions. Compared to methods based on diffusion tensor imaging connectivity and regional gene co-expression, its integration with transcriptional similarity networks is significantly more robust, offering a superior framework for linking genomic processes to MRI-derived phenotypes of cortical structure. Moreover, the MIND network represents a heritable phenotype, with heritability levels comparable to those of established local MRI metrics, such as cortical thickness [17]. This feature provides a critical foundation for identifying the genetic factors underlying individual differences in the structure similarity network. Previous network-based statistical analyses have further revealed that these similarity networks exhibit alterations in whole-brain network properties [18, 19], indicating changes in the hierarchical organization of brain networks. Consequently, structural similarity gradients derived from structure similarity networks are capable of reflecting developmental changes in the cerebral cortex.

As neuroimaging features serve as intermediate phenotypes with stronger theoretical proximity to the genetic underpinnings of LPE, recent advances in imaging transcriptomics have increasingly integrated cortical morphology and functional connectivity abnormalities in LPE patients with spatial gene expression profiles, aiming to unravel the intricate multiscale mechanisms driving this disorder. Recent studies indicate that genetic factors significantly influence the organization of human brain networks, particularly in forming connections that are functionally essential and metabolically demanding [20, 21]. Emerging integrative analyses of transcriptome-connectome interactions provide a pivotal platform for elucidating structural-functional relationships between the microscale transcriptome profile and the macroscale brain network [22–25]. Specifically, the functional architectures of the connectomes are associated with gene expression profiles involving many biological processes such as ion channel activity and oxidative metabolism [26, 27].

Besides, the pathological changes of LPE also involve the serotonergic and non-serotonergic neurotransmitter system [28]. LPE were reported to be related to neurotransmitter disorders, and the potential relationship between these neurotransmitter signatures and macroscopic brain alterations remains to be fully elucidated [6, 29, 30]. Therefore, we speculate that the abnormalities observed in the macroscopic connectome gradients of LPE patients may be associated with neurotransmitters. Elucidating the relationship between the gradient and transcriptome profile can enhance our understanding of the molecular genetic basis of dysfunction in LPE, and provide a more reliable and objective basis for diagnosis and treatment.

In this study, we: (1) aim to confirm that the brain gradients constructed based on the structural similarity network exhibit a pattern of separation and integration; (2) analyze the gradient characteristics of brain regions that differ in patients with LPE and their correlations with symptoms of premature ejaculation; and (3) seek to elucidate the neurobiological mechanisms underlying LPE.

## Materials and methods

### Participants

In this study, we recruited 115 participants between 2022 and 2024, comprising 62 patients with LPE and 53 healthy controls (HC). The study design was explained in detail to all participants, and written informed consent was obtained. All participants completed the following four questionnaires: the International Index of erectile function-5 (IIEF-5) [31], the Premature ejaculation diagnostic tool (PEDT) [32], the Intravaginal ejaculatory latency time (IELT), and the Chinese Index of Premature Ejaculation-5 (CIPE-5) [33]. The study was approved by the Ethics Committee of Nanjing Drum Tower Hospital, Affiliated Hospital of Medical School, Nanjing University, China, and all participants are informed and consented. For more detailed information regarding the participants, please see the Supplementary Materials.

### MRI data acquisition

Imaging was acquired on a 3-T uMR 790 MRI (Shanghai United Imaging Healthcare Co., Ltd.) system with a 32-channel head coil. This study obtained high-resolution 3D T1-weighted imaging brain structural images of each participant. The acquisition parameters for the structural images were as follows: echo time = 2.9 ms; repetition time = 7.1 ms; flip angle = 8°; volume of view = 256 × 256× 176 mm³; slice thickness = 1 mm, artificial intelligence-assisted compressed sensing acceleration factor = 2.22.

### Image processing and MIND structural similarity gradient construction

The MIND method used in this study has been described in this study [16]. Briefly, we describe the definition of MIND as a statistical metric of structural similarity given a surface reconstruction of the cortex. This surface can be described by a set of vertices ${{{{\boldsymbol{v}}}}}_{i}\in \nu$, where each ${{{{\boldsymbol{v}}}}}_{i}$ is a vector of *d* structural features, such as cortical thickness, mean curvature, sulcal depth, surface area, and gray matter volume. These features are automatically generated at the vertex level by FreeSurfer’s *recon-all* command. A cortical Schaefer 2018 parcellation with *400* regions was used in this study [34]. For a given pair of regions $a$ and $b$, we estimate the Kullback–Leibler divergence of ${P}_{b}$ from ${P}_{a}$, denoted as ${D}_{{KL}}({P}_{a}{||}{P}_{b})$. Because Kullback–Leibler divergence is not symmetric, we use a commonly used symmetric version of the metric, computed as follows [35, 36]:

$$D({P}_{a},{P}_{b})={D}_{{KL}}({P}_{a}{||}{P}_{b})+{D}_{{KL}}({P}_{b}{||}{P}_{a})$$

**Fig. 1:**
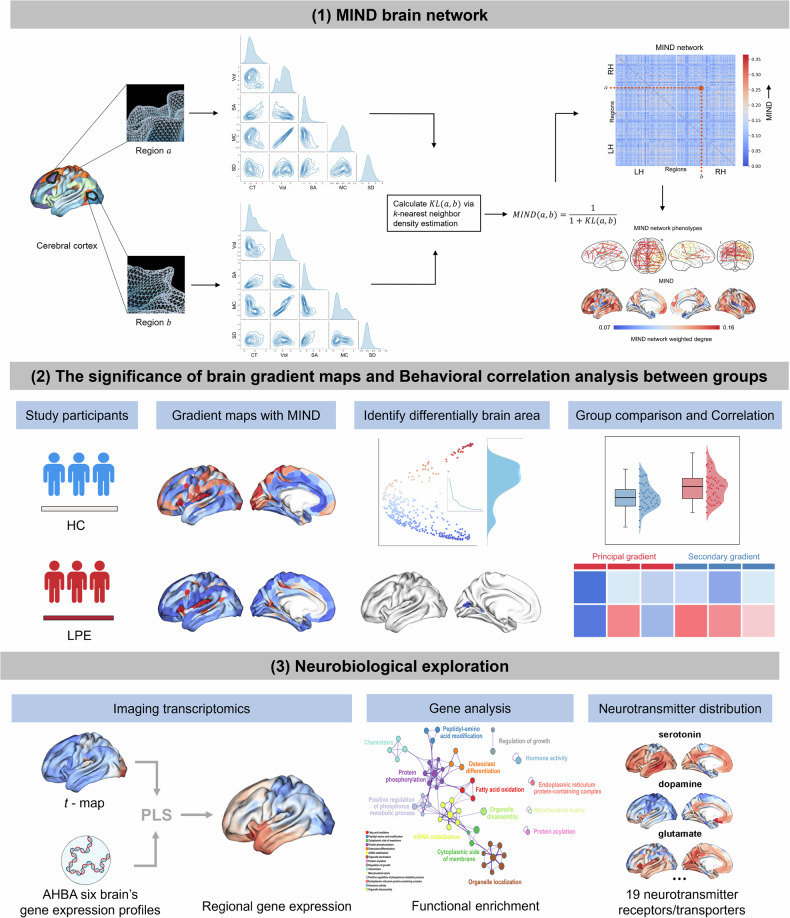
Study overview. (**1**) First, we constructed a cortical surface mesh, which can be described by a set of vertices, each characterized by five structural MRI features: CT, SA, Vol, MC, and SD. To estimate the similarity between cortical regions, we standardized each MRI feature for all vertices and then aggregated all MRI metrics for all vertices within each cortical region (defined by a previous parcellation template) to form a regional multivariate distribution. Next, we used the k-nearest neighbor density algorithm to compile a pairwise distance matrix to estimate the symmetric Kullback–Leibler divergence between each pair of regional multivariate distributions. Finally, we transformed the Kullback–Leibler divergence ${KL}(a,b)$ between regions a and b into an estimated inter-regional MIND similarity, with values ranging from 0 to 1, where higher values indicate greater similarity. The illustrative distributions of regions a and b are presented in a scatter plot matrix, with the diagonal panels showing the marginal univariate distributions of the five structural features and the off-diagonal panels showing the bivariate relationships for each pair. A visualization of a set of average MIND similarity matrices and brain cortical surface maps of two basic MIND network phenotypes - that is, the edges between cortical nodes (here showing the top 2%) and weighted node degrees, calculated by averaging the edge weights for each of the 318 cortical nodes defined by the DK parcellation. (**2**) Based on the MIND network, the structural similarity gradient was calculated for LPE patients and HC to analyze the gradient characteristics of the differential brain regions and their correlations with behavioral measures. (**3**) Based on the Allen Human Brain Atlas, we extracted significant genes associated with the structural gradients of differential brain regions, and subsequently performed gene enrichment analysis and examined neurotransmitter distribution patterns. CT, Cortical thickness; KL, Kullback–Leibler; LPE, Lifelong premature ejaculation; MC, Mean curvature; MIND, Morphological inverse divergence; MRI, Magnetic resonance imaging; SA, Surface area; SD, Sulcal depth; Vol, Volume

The value of $D\left({P}_{a},{P}_{b}\right)$ corresponds to the value of Kullback–Leibler $\left(a,b\right)$ referred to in Fig. 1, which is used for simplicity in the diagram. We define the MIND metric of similarity, bounded between 0 and 1, as follows:

$${MIND}(a,b)=\frac{1}{1+D({P}_{a},{P}_{b})}$$

### The construction of structural similarity gradients

After generating a 400 × 400 structural similarity connectivity matrix based on the MIND network for each participant, the gradient mapping was calculated using the BrainSpace toolbox [34]. We calculated cosine similarity using the MIND network as a similarity measurement. Subsequently, the cosine similarity matrix was input into the diffusion map embedding algorithm, which is a nonlinear dimensionality reduction technique designed to identify gradient components explaining most connectome variances [37]. All the settings were maintained at their default values, with *α* set at 0.5 and *t* at 0 [38].

### The differences between the two groups of structural similarity gradients and correlation analysis

We used independent samples t-tests to assess differences in brain region metrics between the two groups of participants for both the principal and secondary gradients separately. The gradient values of all regions of interest were analyzed for correlations with clinical scale scores using Pearson’s correlation test. A significance threshold of *p* < *0.05* was applied. Multiple comparisons were adjusted using the Bonferroni corrected method.

### Association between structural similarity gradients and cortical gene expression

Transcriptomic data were obtained from the Allen Human Brain Atlas [39, 40]. We introduced a computational framework integrating neuroimaging and genomics. Spatial correlations between the structural similarity gradient (*t*-map) of LPE patients and genome-wide expression patterns were assessed using partial least squares (PLS) regression [41]. Bootstrap resampling (10,000 iterations) assigned robust *Z* scores to gene weights on PLS1, enabling rank-based prioritization of genes with FDR-adjusted significance (*q* < 0.001). Enrichment analysis of these genes identified the 20 most statistically significant gene ontology (GO) biological processes. The top 15 terms were visualized in an interaction network using Cytoscape [42], illustrating functional hierarchies and cross-talk mechanisms. The network was constructed using Metascape [43].

### Association between structural similarity gradients and brain-wide neurotransmitter distribution

We further investigated the relationship between the structural similarity gradient and neurotransmitter distribution patterns. To examine the spatial correspondence between the structural similarity gradient map and neurotransmitter distribution, we computed the Spearman correlation between the structural similarity gradient map of LPE patients and individual neurotransmitter receptor/transporter density maps. We accounted for spatial autocorrelation using a spin test—a permutation method that rotates brain data on a sphere to preserve spatial covariance, producing a valid null distribution. Statistical significance (*p*_spin_) was assessed against 10,000 rotations, and *p*-values were Bonferroni-corrected for multiple comparisons. Neurotransmitter data acquisition details are in the Supplementary Materials.

### Methodical framework

The methodological framework of this study is illustrated in Fig. 1. First, we constructed the MIND network from three-dimensional T1-weighted imaging structural data. Next, we derived the structural similarity gradient from the MIND network for each participant. We then computed the brain structural gradients for both groups, analyzed the characteristics of structural gradients in differential brain regions, and examined their correlations with behavioral traits. Finally, using the Allen Human Brain Atlas [39, 40], we extracted significant genes associated with these differential brain region gradients, performed gene enrichment analysis, and examined the distribution characteristics of neurotransmitters.

## Results

### Demographics

Between-group statistical comparison showed no significant difference in the IIEF-5 score of LPE patients (*p* = 0.263). The PEDT scores (*p* < 0.001) and the CIPE-5 scores (*p* < 0.001) were significantly lower in LPE patients compared to the control group. The IELT was significantly shorter in LPE patients (*p* < 0.001). Furthermore, no significant differences were observed between the two groups in terms of age, marital status, or educational level (all *p* ≥ 0.273), as detailed in Table 1.

**Table 1:** Demographic and clinical characteristics used in the analysis

	LPE patients (*n* = 62)	HC (*n* = 53)	Statistic	*p*
Age, years	28.85 ± 4.99	29.74 ± 4.91	-0.578	0.565
Marital status, *n* (%)			0.321	0.571^#^
Single	29 (46.77%)	22 (41.51%)		
Married	33 (53.23%)	31 (58.49%)		
Education level, *n* (%)			2.597	0.273^#^
Elementary	10 (16.13%)	5 (9.43%)		
High school	20 (32.26%)	13 (24.53%)		
University	32 (51.61%)	35 (66.04%)		
IIEF-5 score	22.71 ± 1.63	22.49 ± 2.68	-1.124	0.263
CIPE-5 score	8.98 ± 2.54	22.28 ± 2.21	-29.421	< 0.001
PEDT score	14.73 ± 3.65	3.68 ± 2.53	18.399	< 0.001
IELT (in min)	1.06 ± 0.71	11.53 ± 7.10	-11.446	< 0.001

Data from questionnaires were reported as mean scores (mean) and standard deviations (SD) for both the LPE and HC groups. Statistical analysis included chi-squared test when indicated by #, and two-sample t-test were employed in the absence of #

*CIPE-5* Chinese index of premature ejaculation-5, *HC* Healthy controls, *IELT* Intravaginal ejaculatory latency time, *IIEF-5* International index of erectile function-5, *LPE* Lifelong premature ejaculation, *PEDT* Premature ejaculation diagnostic tool

### Structural similarity gradients in LPE and HC

**Fig. 2:**
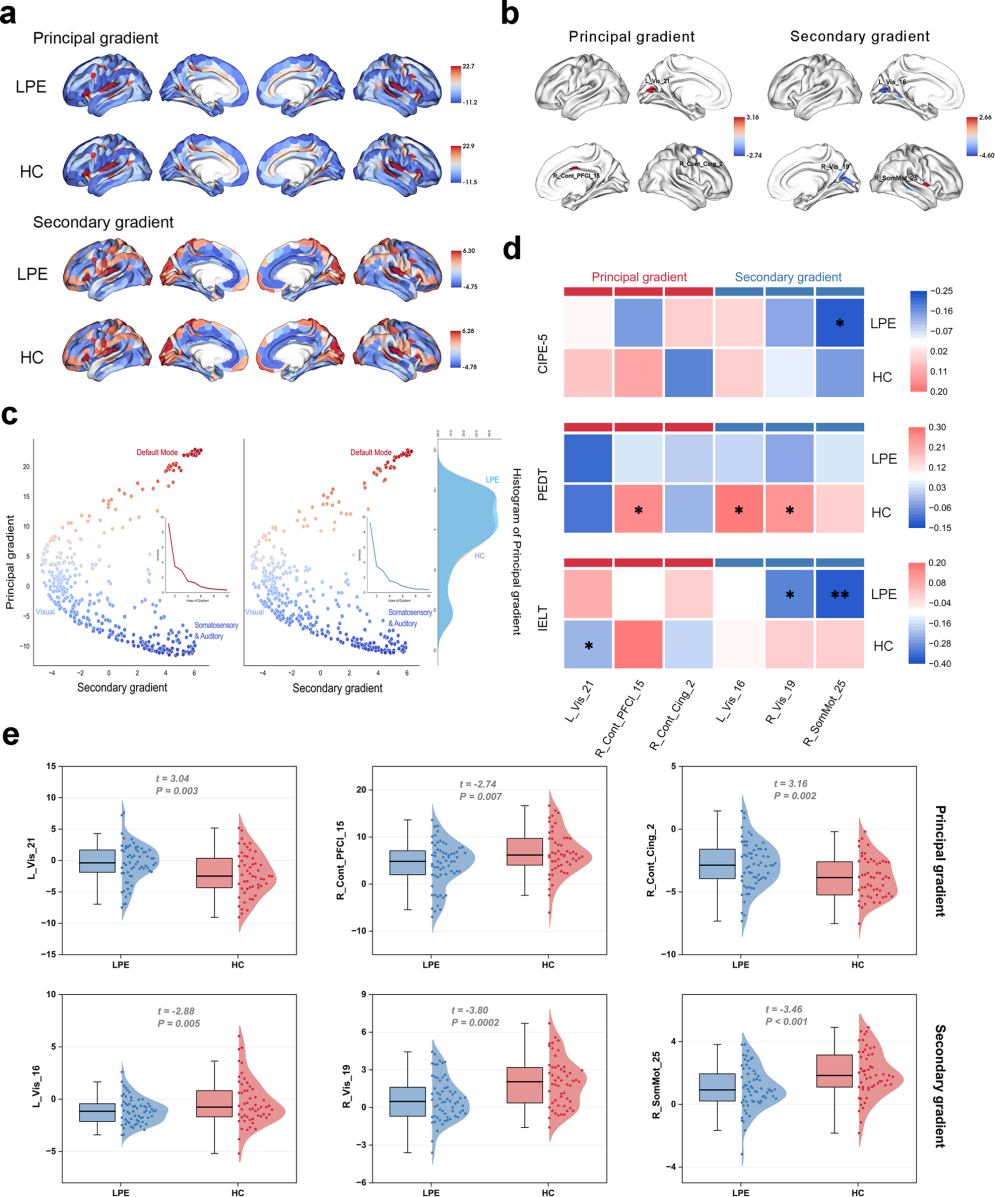
The significance of brain gradient maps and Behavioral correlation analysis between groups. **a** The group-average cortical connectome gradient patterns respectively revealed the principal and secondary structural similarity gradients of LPE patients and HC. **b** In LPE patients, the principal gradient increased in the left visual cortex and the right prefrontal region (Vis_21, Cont_PFCl_15, *p* < 0.001), and decreased in the right cingulate gyrus region (Cont_Cing_2, *p* < 0.001). The secondary gradient decreased in the right somatosensory cortex (SomMot_25, *p* < 0.001), and decreased in the bilateral visual cortex regions (Left_Vis_16, Right_Vis_19, *p* < 0.001). **c** Scatter plots of the principal and secondary structural similarity gradients for the LPE patients and HC. The *y*-axis represents the principal gradient, showing the changes from the default mode network to the somatomotor and auditory networks. The *x*-axis represents the secondary gradient, which separates the visual network from the somatomotor and auditory network. **d** In LPE patients, the CIPE-5 score exhibited a significant negative correlation with the secondary gradient of the right SomMot_25 (*r* = -0.221, *p* = 0.038), while the IELT score showed significant negative correlations with the secondary gradients of the right Vis_19 (*r* = -0.31, *p* = 0.014) and right SomMot_25 (*r* = -0.369, *p* = 0.003). In the HC group, the PEDT score was positively correlated with both the principal and secondary gradients of the right Cont_PFCl_15 (*r* = 0.268, *p* = 0.043), left Vis_16 (*r* = 0.273, *p* = 0.048), and right Vis_19 (*r* = 0.245, *p* = 0.045). Additionally, the IELT score was negatively correlated with the principal gradient of the left Vis_21 (*r* = -0.232, *p* = 0.045). After Bonferroni correction, only the right SomMot_25 was significantly negatively correlated with the IELT score (*r* = -0.369, *p* = 0.003). **e** Independent sample t-tests were performed to compare the structural similarity gradients between LPE patients and HC group. After Bonferroni correction, brain regions with *p*-values less than 0.05 were identified, and their gradient conditions were visualized. CIPE-5, Chinese index of premature ejaculation-5; HC, Healthy controls; IELT, Intravaginal ejaculatory latency time; IIEF-5, International index of erectile function-5; LPE, Lifelong premature ejaculation; PEDT, Premature ejaculation diagnostic tool

The principal (27.65 ± 6.54%) and the secondary (14.76 ± 3.64%) gradients explained the most variance of the connectomes (Fig. 2a). Similar to the canonical distribution, both LPE patients and the HC group exhibited a distinct transition from a unimodal system to a transmodal system in the principal gradient. And principal networks were separated in the secondary gradient, with the visual network localized on one end and the somatomotor network on the other end.

### Differential brain regions between LPE and HC in structurally similar gradients

We further compared the principal and secondary gradients between groups at the region-of-interest level. After Bonferroni corrected, brain regions with *p*-values less than 0.001 were identified, and their gradient conditions were visualized (Fig. 2b). The results showed that in LPE patients, the principal gradient increased in the left visual cortex and the right prefrontal region (Vis_21: *p* = 0.003; Cont_PFCl_15, *p* = 0.007, Bonferroni corrected), and decreased in the right cingulate gyrus region (Cont_Cing_2: *p* = 0.002, Bonferroni corrected). The secondary gradient decreased in the right somatosensory cortex (SomMot_25: *p* < 0.001, Bonferroni corrected), and decreased in the bilateral visual cortex regions (Left_Vis_16: *p* = 0.005; Right_Vis_19: *p* < 0.001, Bonferroni corrected) (Fig. 2e).

### The correlation between structural similarity gradient’s features and clinical variables

Pearson correlation analysis was conducted to evaluate the correlations between changes in structural similarity gradient scores and clinical variables. In the HC group, the PEDT score was positively correlated with both the principal and secondary gradients of the right Cont_PFCl_15 (*r* = 0.268, *p* = 0.043), left Vis_16 (*r* = 0.273, *p* = 0.048), and right Vis_19 (*r* = 0.245, *p* = 0.045). Additionally, the IELT score was negatively correlated with the principal gradient of the left Vis_21 (*r* = -0.232, *p* = 0.045). In LPE patients, the CIPE-5 score exhibited a significant negative correlation with the secondary gradient of the right SomMot_25 (*r* = -0.221, *p* = 0.038), while the IELT score showed significant negative correlations with the secondary gradients of the right Vis_19 (*r* = -0.31, *p* = 0.014) and right SomMot_25 (*r* = -0.369, *p* = 0.003). After Bonferroni correction, only the right SomMot_25 (*r* = -0.369, *p* = 0.003) was significantly negatively correlated with the IELT score (Fig. 2d).

### Association between structural similarity gradient and cortical gene expression

**Fig. 3:**
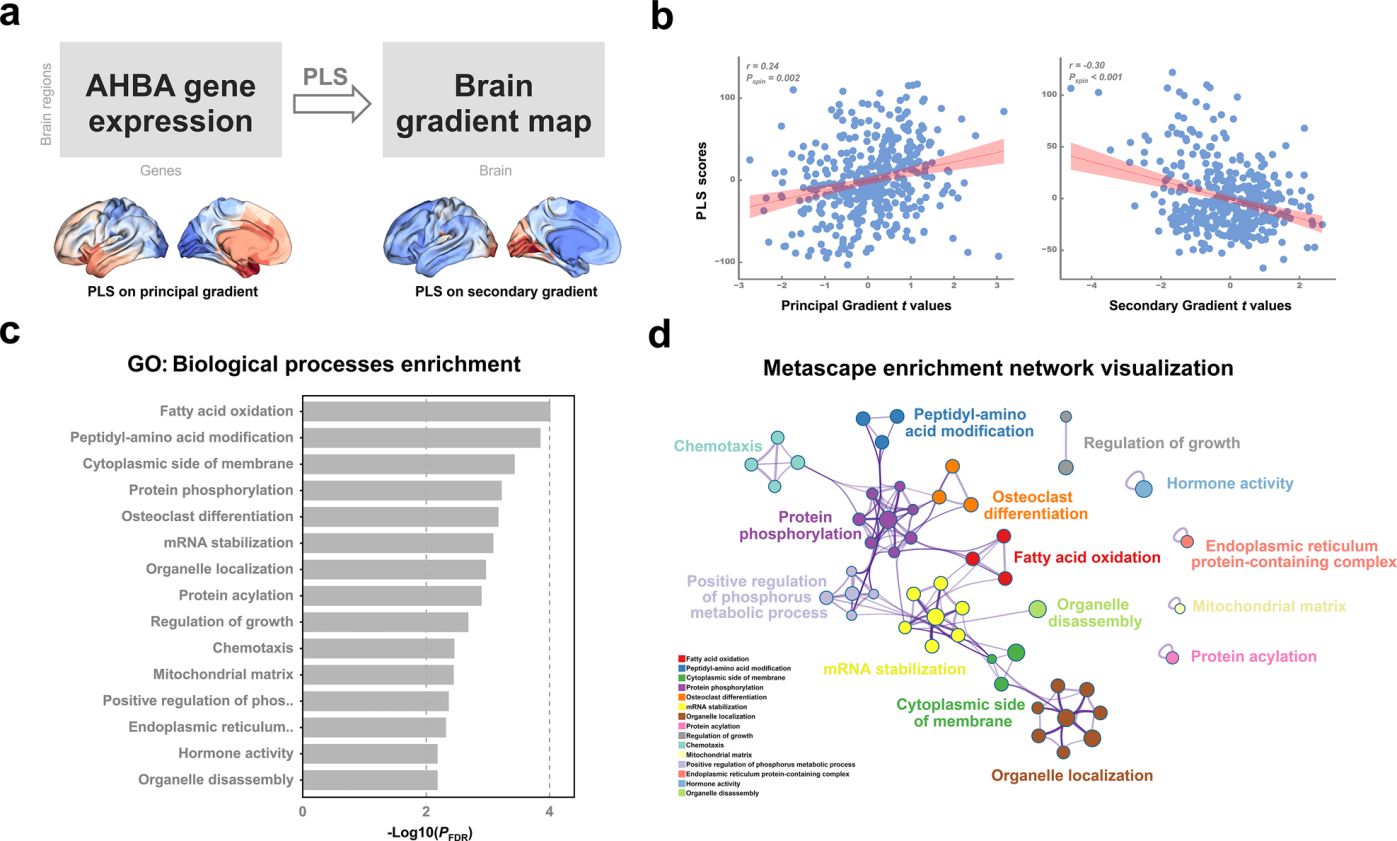
Gene expression profiles related to the structural similarity gradient map. **a** Using Allen Human Brain Atlas-derived bilateral-hemispheric transcriptomic data, PLS regression was used to examine the spatial association between the structural similarity gradient map (*t*-values) and gene expression levels within the bilateral hemisphere. **b** The PLS components are spatially correlated with the principal and secondary structural similarity gradient map. **c** Top 15 significantly enriched GO biological processes identified through Metascape analysis. **d** Visualization of enrichment networks using Cytoscape. In this visualization, each node corresponds to an enriched term. The size of the node reflects the number of input genes associated with that term, while the color indicates the cluster to which it belongs (*i.e.*, nodes with the same color are part of the same cluster). GO, Gene ontology; PLS, Partial least squares

We obtained a brain gene expression matrix based on the same Schaefer parcellation (*n* = 400). In addition, genes whose similarity across donors fell below a threshold (*r* < 0.2) were further removed, leaving a total of 9,138 genes for the following analysis. PLS regression was used to examine the spatial association between structural similarity gradient *t* map and gene expression levels for all 9,138 genes across the 400 regions across both hemispheres (Fig. 3a). We revealed that the first PLS component exhibited significant spatial correlations with the principal and secondary structural similarity gradient *t*-maps (PLS _principal_: *r* = 0.24, *p*_spin_ = 0.002; PLS _secondary_: *r* = -0.30, *p*_spin_ < 0.001; Fig. 3b). We ranked the genes according to their weights (*Z* scores of PLS loadings) to each PLS component by bootstrapping. The list of genes with an FDR *p* < 0.001 (both positive *Z* score and negative *Z* score) was extracted for enrichment analysis using Metascape. Enrichment analysis revealed the top 20 most significant GO biological processes, including “Hormone activity”, “protein phosphorylation”, “mRNA stability”, and “fatty acid oxidation”(Fig. 3c). To better illustrate the connections between these enriched terms, a selection of them was visualized as a network graph using Cytoscape (Fig. 3d). We similarly repeated this process for secondary gradient (details are provided in the Supplementary Materials).

### Association between structural similarity gradient and brain-wide neurotransmitter distribution

**Fig. 4:**
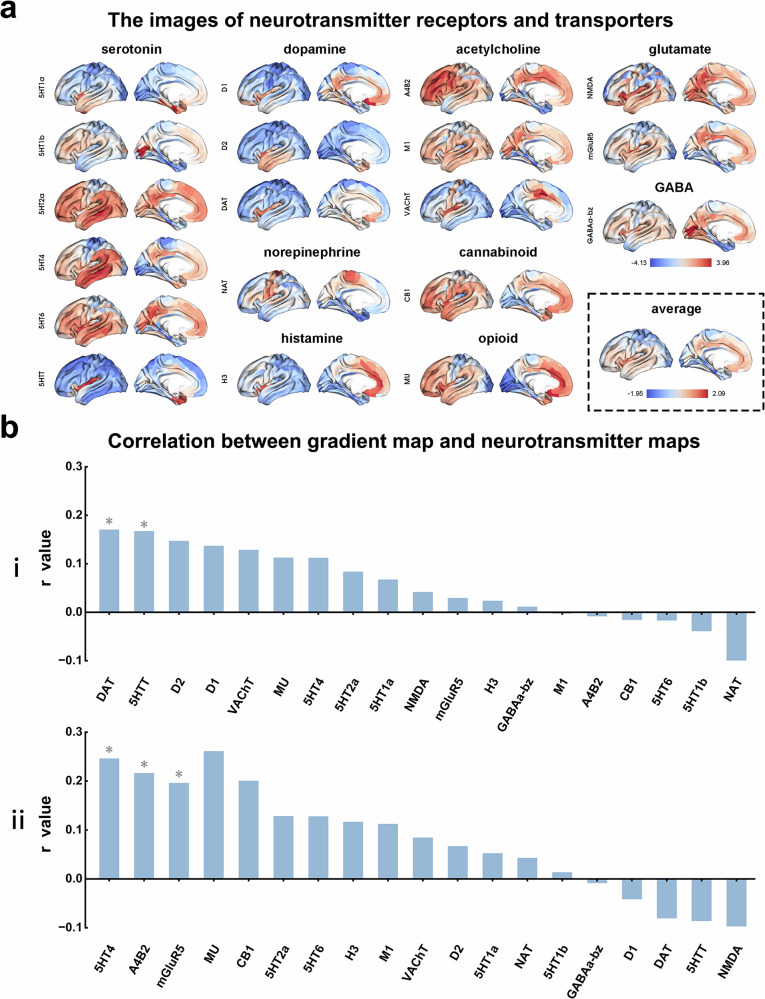
Neurotransmitter receptors/transporters density maps related to the structural similarity gradient map. **a** Neurotransmitter receptor/transporter density maps are obtained from positron emission tomography images over 1,200 healthy individuals, including 19 neurotransmitter receptor/transporter density maps (including dopamine, norepinephrine, serotonin, acetylcholine, glutamate, γ-aminobutyric acid (GABA), histamine, endocannabinoid, and opioid), across 9 different neurotransmitter systems. **b** Spatial correlation between the principal (**i**) and secondary (**ii**) structural similarity, structural similarity gradient map, and neurotransmitter receptors/transporters density maps. Asterisk indicates significant correlation after multiple comparisons correction (*p*_spin_ < 0.001)

We found that the principal structural similarity gradient had significant spatial correlations with the neurotransmitter density maps of the dopamine transporter (*r* = 0.17, *p*_spin_ < 0.001) and serotonin transporter (5HTT: *r* = 0.17, *p*_spin_ < 0.001), while in the secondary gradient, such correlations also occurred in the neurotransmitter density maps of the serotonin transporter (5HT4: *r* = 0.25, *p*_spin_ < 0.001), nicotinic acetylcholine receptor (α4β2: *r* = 0.22, *p*_spin_ < 0.001) and glutamate (mGluR5: *r* = 0.20, *p*_spin_ < 0.001) (Fig. 4b).

## Discussion

In this study, we provide the first evidence for abnormal structural similarity gradients in patients with LPE and investigate their associations with transcriptomic and neurotransmitter systems. Subjects exhibited a canonical pattern in the gradient. In LPE patients, the principal gradient was elevated in the left visual cortex and right prefrontal region, and reduced in the right cingulate gyrus. The secondary gradient was reduced in the right somatosensory cortex, as well as in the bilateral visual cortex regions. Our connectome–transcriptome association analysis identified associations between LPE-related gradient alterations and gene pathways related to hormone activity, protein phosphorylation, mRNA stability, and fatty acid oxidation. Furthermore, we demonstrated a relationship between neurotransmitter receptor/transporter distribution—including systems such as serotonin and dopamine—and gene expression profiles in LPE patients. These findings offer novel insights into the neurobiological mechanisms underlying LPE and suggest how macrostructural brain changes are linked to molecular and cellular processes.

Studies have demonstrated that MIND is driven by structural features that reliably distinguish cortical areas, and that the structural architecture of the connectome mirrors the organization of the brain’s gene co-expression network [16, 26, 27]. In recent years, in-depth analyses of brain structure and network hierarchy have further reinforced the overarching hierarchical organization of the connectome. Classical neuroanatomical findings and neuroimaging research have provided robust evidence supporting this architecture, thus laying a critical foundation for understanding cerebral information processing [38, 44]. In our study, we observed that the structural similarity gradient constructed using the MIND network closely resembles the gradient derived from functional connectivity data. We found that the structural gradient revealed a continuous axis of connectivity variation along the principal gradient, originating from the default mode network and extending toward cross-modal regions, primarily encompassing the visual, somatosensory, and auditory networks. This similarity may be because structural data better reflect the developmental principles of cortical organization. Furthermore, the structural similarity gradient, which captures gradual transitions in the connectome, may provide a unified framework for explaining the diverse behavioral phenotypes observed in LPE patients and for linking anomalies across macroscale functional networks.

Our findings indicate that the connectome gradient patterns in LPE patients are abnormal, and the alteration of clinical phenotypes may be predominantly driven by the transition from unimodal to cross-modal systems and the suppression of principal network separation. The visual network and somatomotor network play critical roles in LPE patients. The visual network is primarily responsible for processing sex-related visual stimuli. Correlation analysis between the gradient and clinical scores suggests that patients with premature ejaculation may exhibit heightened sensitivity to visual sexual stimuli, thereby accelerating sexual arousal and the ejaculation reflex. The functional connectivity between the visual cortex and regions such as the insula and anterior cingulate cortex during exposure to sexual stimuli differs from that in healthy individuals (*e.g.*, over-activation or enhanced connectivity) [10]. When sensory input signals from the penis and other genital organs are over-represented or abnormally activated in the principal somatosensory cortex (S1), these signals are transmitted to the principal motor cortex (M1) and supplementary motor area (SMA) to participate in the inhibition of voluntary movement [45, 46]. This process may disrupt the higher-level central control of pelvic floor muscles, leading to an inability to delay the ejaculation reflex [47, 48]. Given that the cerebral cortex serves as the higher-order control center for ejaculation-related activities, its abnormal development directly impacts the macroscopic structural gradients.

Our connectome-transcriptome association analysis revealed significant correlations between LPE-related connectome gradient alterations and multiple biological pathways. Notably, the “hormone activity” pathway shows a complex relationship with premature ejaculation pathophysiology [3], reflecting integrated physiological system functioning. Testosterone, the primary male sex hormone, significantly extends intravaginal ejaculation latency time in individuals with premature ejaculation [49]. Hormonal imbalances may disrupt this equilibrium, suggesting future research should focus on gene-hormone-environment interactions to advance personalized treatments. 5-hydroxytryptamine (5HT), a key neurotransmitter in premature ejaculation, regulates ejaculation inhibition and emotional processes. Its synthesis depends on “protein phosphorylation” and “mRNA stability” pathways. Tryptophan hydroxylase, the rate-limiting enzyme for 5-HT synthesis, is regulated by phosphorylation [50]. Enhanced mRNA stability increases tryptophan hydroxylase synthesis, while reduced stability decreases it. mRNA stability can also indirectly influence tryptophan hydroxylase phosphorylation, affecting enzymatic activity [51]. Additionally, “fatty acid oxidation,” an essential energy metabolism pathway, produces acetyl-CoA, which may modulate tryptophan hydroxylase activity and impact 5-HT synthesis. Based on these findings, we hypothesize that decreased serotonin levels correlate with increased body lipid levels, linking principal premature ejaculation to metabolic disorders such as obesity, which offers valuable directions for future investigations. These pathways likely interact intricately in serotonin metabolism and brain region function, where dysfunction may lead to structural and functional changes, altering physiological regulation and providing new insights into LPE’s neurobiological basis.

Subsequently, we further elucidated the regulatory mechanisms of neurotransmitter systems underlying the observed gradient changes in structural similarity in LPE patients. Previous studies have also reported alterations in central nervous system neurotransmitters in LPE patients [30]. Neurotransmitters can regulate specific brain functions through excitatory and inhibitory pathways, including serotonergic, dopaminergic, and cholinergic systems that are involved in the regulation of ejaculation [28, 52–54]. In our study, the principal gradient changes showed a positive correlation with 5-HT and dopamine transporter expression, suggesting that these neurotransmitter systems may enhance the brain’s information transmission capacity through excitatory mechanisms. In contrast, the reduced expression of 5-HT4, α4β2, and mGluR5 in the secondary gradient implies a potential inhibitory effect on information transmission. Taken together, although multiple neurotransmitter systems in LPE pathology, they appear to modulate distinct aspects of brain information processing through diverse mechanisms, ultimately contributing to the maintenance of neural stability.

There are several limitations in the present study. First, our study did not account for subcortical structures, which could potentially introduce bias, particularly given the critical role of subcortical hubs in neurotransmitter system dysfunction. Second, investigating longitudinal changes in the principal gradients during disease progression is important, as this may enhance our understanding of the topological organization in LPE patients. Third, the collection and analysis of neurobiological data will be crucial for confirming LPE-related gradient alterations in future research. Finally, we utilized gene expression data from six healthy brains, obtained from donors with varying ages, ethnicities, and genders, to explore the relationship between the *t*-map of LPE gradients and gene expression, which may have led to variance in the gene expression data.

In conclusion, this study has characterized the brain structural gradient alterations in LPE patients, identified gene-enriched biological pathways implicated in pathological processes, and examined the distribution patterns of neurotransmitter receptors and transporters. These findings provide important insights into the neuroimaging and neurobiological mechanisms underlying LPE.

## Supplementary information

**Additional file 1: Supplementary Fig. S1.** Gene expression profiles related to secondary structural similarity gradient map.

## Data Availability

The datasets used and/or analyzed during the current study are available from the corresponding author on reasonable request.

## Abbreviations

5-HT: 5-hydroxytryptamine
CIPE-5: Chinese index of premature ejaculation-5
GO: Gene ontology
HC: Healthy controls
IELT: Intravaginal ejaculatory latency time
IIEF-5: International index of erectile function-5
LPE: Lifelong premature ejaculation
MIND: Morphological inverse divergence
MRI: Magnetic resonance imaging
PEDT: Premature ejaculation diagnostic tool
PLS: Partial least squares
