# Supplementary material for: Assessing neurobiology of lifelong premature ejaculation through brain MRI structural similarity gradient
Source: Eur Radiol Exp. 2025 Dec 18;9:119. doi: 10.1186/s41747-025-00661-3 (PMC12715095; doi:10.1186/s41747-025-00661-3)
Supplement: Supplementary file 1 — Additional file 1: Supplementary Fig. S1. Gene expression profiles related to secondary structural similarity gradient map. [file 41747_2025_661_MOESM1_ESM.pdf]

Assessing neurobiology of lifelong premature ejaculation through brain MRI structural similarity gradient

ELECTRONIC SUPPLEMENTARY MATERIAL

Participants' Supplementary Information

Patients with lifelong premature ejaculation (LPE) were diagnosed according to ISSM guidelines: (1) ejaculation that always or nearly always occurs within approximately one minute of vaginal penetration; (2) the inability to delay ejaculation during nearly all instances of vaginal penetration; and (3) negative personal consequences such as distress, disturbance, frustration, and/or the avoidance of sexual intimacy. We excluded individuals with erectile dysfunction, genitourinary tract infections, systemic or neurological conditions, or self-reported psychological disorders. The HC group consisted of individuals who self-reported intravaginal ejaculatory latencies of more than three minutes. The IELT was measured during a 4-week baseline period, during which both patients and healthy men were asked to engage in sexual intercourse at least four times. All participants were in a stable relationship with the same, non-pregnant, sexually active partner for at least one year. Prior to participation in the study, all eligible participants underwent a comprehensive andrological diagnostic workup, including a detailed medical history assessment, physical examination, and hormonal evaluation to rule out other potential causes of reported sexual dysfunction. Furthermore, we excluded participants with structural abnormalities that could cause cognitive impairment, as identified by conventional MRI. All participants were recruited from the clinic of Andrology Department in the Affiliated Drum Tower Hospital of Nanjing University, Nanjing, China.

The acquisition methods of neurotransmitters and related information

A comprehensive cortical profile of neurotransmitter receptor densities was previously obtained from PET images of more than 1200 healthy individuals across multiple studies (https://github.com/netneurolab/hansen_receptors, version 1, July 2022). A total of 19 distinct neurotransmitter receptor and transporter maps[1], spanning nine neurotransmitter systems—including dopamine, norepinephrine, serotonin, acetylcholine, glutamate, GABA, histamine, cannabinoid, and opioid—were obtained and parcellated into 400 cortical regions using the same atlas as the structural similarity gradient map.

Association between the secondary structural similarity gradient and cortical gene expression

We similarly repeated this process for secondary structural similarity gradient (Supplementary Fig. S1). Enrichment analysis revealed the top 15 most significant Gene Ontology biological processes, such as “lung development,”“Regulation of actin cytoskeleton organization,” and “Positive regulation of calcium-mediated signaling”.

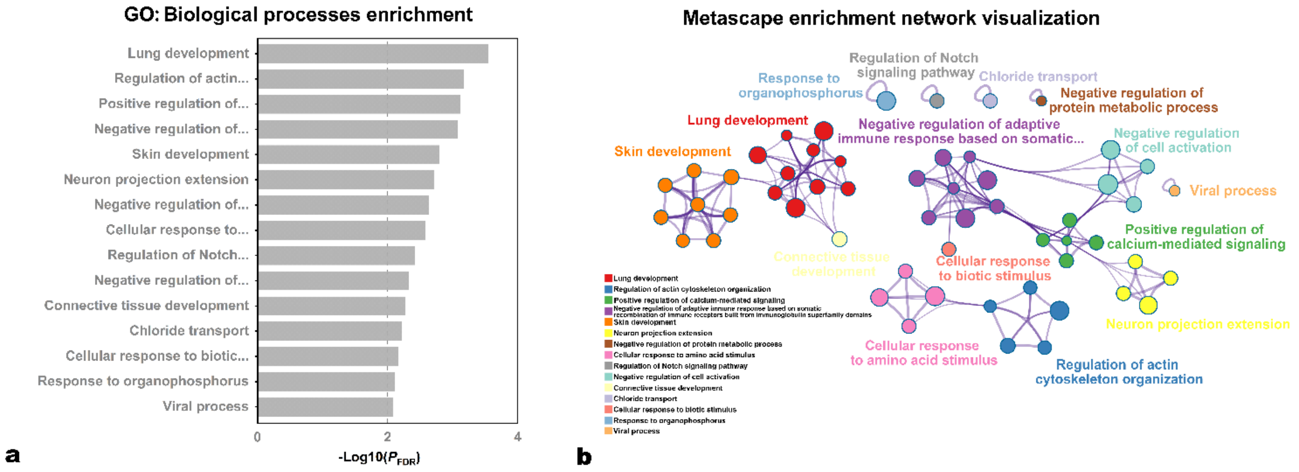

Supplementary Fig.S1 | Gene expression profiles related to secondary structural similarity gradient map

(a) Top 15 significant enriched Gene Ontology biological processes identified through Metascape analysis. (b) Visualization of enrichment networks using Cytoscape. In this visualization, each node corresponds to an enriched term. The size of the node reflects the number of input genes associated with that term, while the color indicates the cluster to which it belongs (i.e., nodes with the same color are part of the same cluster).

Reference

1. Hansen J Y, Shafiei G, Markello R D et al (2022) Mapping neurotransmitter systems to the structural and functional organization of the human neocortex. Nat Neurosci 25:1569-1581. https://doi.org/10.1038/s41593-022-01186-3.

Chinese Index of Premature Ejaculation-5

Q1. How long from intromission to ejaculation?

1. Too short (< 30 sec)
2. Very short (< 1 min)
3. Short (< 2 min)
4. Often short (< 3 min)
5. Not short (< 3 min)

Q2. Can you prolong the intercourse time?

1. Very difficult
2. Always difficult
3. Difficult
4. Seldom difficult
5. Not difficult

Q3. Your sexual satisfaction?

1. Very dissatisfied
2. Always dissatisfied
3. Generally satisfied
4. Often satisfied
5. Always satisfied

Q4. Your partner's sexual satisfaction?

1. Very dissatisfied
2. Always dissatisfied
3. Generally satisfied
4. Often satisfied
5. Always satisfied

Q5. Do you feel anxiety, depression or stress in sexual activity?

1. Always
2. Often
3. Half of time
4. Seldom
5. Almost never

International Index of Erectile Function-5

Over the past six months:

Q1. How do you rate your confidence that you could get and keep an erection?

1. Very low
2. Low
3. Moderate
4. High
5. Very high

Q2. When you had erections with sexual stimulation, how often were your erections hard enough for penetration?

1. Almost never/never
2. A few times (much less than half the time)
3. Sometimes (about half the time)
4. Most times (much more than half the time)
5. Almost always/always

Q3. During sexual intercourse, how often were you able to maintain your erection after you had penetrated (entered) your partner?

1. Almost never/never
2. A few times (much less than half the time)
3. Sometimes (about half the time)
4. Most times (much more than half the time)
5. Almost always/always

Q4. During sexual intercourse, how difficult was it to maintain your erection to completion of intercourse?

1. Extremely difficult
2. Very difficult
3. Difficult
4. Slightly difficult
5. Not difficult

Q5. When you attempted sexual intercourse, how often was it satisfactory for you?

1. Almost never/never
2. A few times (much less than half the time)
3. Sometimes (about half the time)
4. Most times (much more than half the time)
5. Almost always/always

Premature Ejaculation Diagnostic Tool

Q1. How difficult is it for you to delay ejaculation?

1. Not difficult at all
2. Somewhat difficult
3. Moderately difficult
4. Very difficult
5. Extremely difficult

Q2. Do you ejaculate before you want to?

1. Almost never or never 0%
2. Less than half the time 25%
3. About half the time 50%
4. More than half the time 75%
5. Almost always or always 100%

Q3. Do you ejaculate with very little stimulation?

6. Almost never or never 0%
7. Less than half the time 25%
8. About half the time 50%
9. More than half the time 75%
10. Almost always or always 100%

Q4. Do you feel frustrated because of ejaculating before you want to?

11. Not at all
12. Slightly
13. Moderately
14. Very
15. Extremely

Q5. How concerned are you that your time to ejaculation leaves your partner sexually unfulfilled?

1. Not at all
2. Slightly
3. Moderately
4. Very
5. Extremely
